# PM_10_ Increases the Daily Outpatient Visits for Asthma: A Time Series Analysis from 2013–2016 in Shanghai, China

**Published:** 2019-08

**Authors:** Xiaolin LIU, Xianfeng ZHOU, Juzhong KE, Hua QIU, Kang WU, Xiaonan WANG, Siyu YU, Zhitao LI, Tao LIN, Xiaonan RUAN, Li PENG

**Affiliations:** 1. Department of Chronic Diseases Control and Prevention, Shanghai Pudong New Area Center for Disease Control and Prevention, Pudong Institute of Preventive Medicine, Fudan University, Pudong New Area, Shanghai, China; 2. Laboratory of Meteorology and Health, Shanghai Meteorological Service, Shanghai, China

## Dear Editor-in-Chief

According to WHO, there are as many as 300 million people suffering from asthma and about 250,000 annual deaths caused by asthma worldwide(1). By some estimates, there may be an additional 100 million patients with asthma by 2025(2). Although asthma can be alleviated spontaneously or through medication, there is no effective treatment that can completely cure the disease (3). This leads to a huge disease burden. Asthma accounts for 1% of all disability-adjusted life years (DALYs) lost globally (2) and ranks 22^nd^ among causes of DALYs loss (1).

The annual medical cost for asthma patients age 14 or older in Beijing was 88 million dollars (4). Annual costs of asthma in the UK were at least 145.8 million dollars in 2011–2012 (5).

During the last decade, fast economic development and social industrialization have caused serious air pollution and changes in weather conditions. Prevention and control of air pollution is a major challenge in China in particular. Increasing evidence has shown that meteorological factors such as temperature and air pollutants such asPM_2.5_, particulate matter less than 10 microns (PM_10_), and ozone (O_3_) could affect the mortality and morbidity of asthma in both children and adults(6).

The objective of this study was to evaluate the association between air pollutants (PM_2.5_, PM_10_, NO_2_, SO_2_, CO and O_3_) and outpatient visits for asthma in Shanghai, China, using advanced analytic methods to control potential confounders. The association between meteorological conditions and outpatient visits for asthma was also explored.

Data of outpatient visits for asthma and demographic characteristics of patients were collected from 10 large hospitals in Pudong New Area, Shanghai between Jan 2013 and Dec 2016. Air pollution data were obtained from the database of the Shanghai Environmental Monitoring Center (SEMC), and meteorological data were obtained from the Shanghai Meteorological Bureau. A generalized additive model was applied to estimate the magnitudes of associations between risk factors and daily outpatient visits for asthma, and to draw statistical inference.

For meteorological factors, daily average temperature lag2 (previous second day concentration’s reading), daily average wind speed lag07 (8-day moving average of readings from the current and previous 7 d) and daily cumulative rainfall volume lag0 (current day’s reading) were statistically related to outpatient visits for asthma. The *logRR* for outpatient visits for asthma was high at about 20 °C for average temperature lag2. A possible explanation for this observation is that a warm weather at about 20 °C and high humidity in Shanghai is the perfect condition for microorganisms to breed, and easily have a lot of pollen and other allergens, which results in increased exposure to outdoor allergens and induces asthma attacks. The risk of asthma attack was generally reduced with the increase of daily average wind speed lag07 and daily cumulative rainfall volume lag0. Acceleration of daily average wind speed and increase in daily cumulative rainfall volume are beneficial to the spread of air pollutants, especially suspended particulates, decreasing their concentration, which is known to reduce asthma attacks. After controlling meteorological factors, which affect the spread of air pollutants and change their concentration, we found PM_10_ lag0 (current day’s reading) was associated with outpatient visits for asthma. Outpatient visits for asthma would increase 1.052 times (95% *CI*: 1.016,1.089, *P*=0.0049) with 1μg/m^3^ increase of PM_10_ lag0 When PM_10_ lag0 concentration was in the 30.1 to 100μg/m^3^ range, and outpatient visits would increase 1.071 times (95% *CI*: 1.008,1.138, *P*=0.0261) when PM_10_ was in the range of 150.1 to 200μg/m^3^. Our results indicated that low concentration of PM_10_could also exacerbate asthma symptoms; even PM_10_ concentration was lower than China and WHO limits.

Asthma patients should strengthen their awareness of disease prevention when there is PM_10_ pollution or temperature is suitable for the pollen and microorganisms increase. Preventative measures include reducing indoor and outdoor air pollution, such as equipping air purifiers at home or wearing a mask when you are outdoors, reducing chance of stay outdoors when temperature is warm in spring.
